# *In vitro* pharmacodynamics of nemonoxacin and other antimicrobial agents against *Mycoplasma pneumoniae*

**DOI:** 10.1128/spectrum.02431-23

**Published:** 2023-11-17

**Authors:** Na Wang, Yuancheng Chen, Xingyi Qu, Xingchen Bian, Jiali Hu, Xiaogang Xu, Li Xiao, Yang Liu, Jing Zhang

**Affiliations:** 1 Institute of Antibiotics, Huashan Hospital, Fudan University, Shanghai, China; 2 Key Laboratory of Clinical Pharmacology of Antibiotics, Shanghai, China; 3 National Health Commission & National Clinical Research Center for Aging and Medicine, Huashan Hospital, Fudan University, Shanghai, China; 4 Department of Medical Oncology, Shanghai Cancer Center, Fudan University, Shanghai, China; 5 Phase I Unit, Huashan Hospital, Fudan University, Shanghai, China; 6 Department of Medicine, University of Alabama at Birmingham, Birmingham, Alabama, USA; Children's National Hospital, George Washington University, Washington, DC, USA

**Keywords:** nemonoxacin, *Mycoplasma pneumoniae*, pharmacodynamics, non-fluorinated quinolone

## Abstract

*Mycoplasma pneumoniae* is the causative agent of respiratory infections in children and adults. Macrolide still plays a significant role in treating infections by *M. pneumoniae*. However, macrolide-resistant *M. pneumoniae* (MRMP) was encountered, causing more severe or prolonged disease worldwide from the early 2000s. Nemonoxacin is a newly developed C-8-methoxy non-fluorinated quinolone having broad-spectrum activity against gram-positive, gram-negative, and atypical pathogens *in vitro*, as well as general safety and well tolerance *in vivo*. This study aimed to assess the killing kinetics of nemonoxacin and other antimicrobial agents against MRMP and macrolide susceptible *M. pneumoniae* (MSMP) by static time-kill curves. In addition, the pharmacodynamic analyses of these drugs were determined by the sigmoid *E*_max_ model. The static time-kill curves indicated that nemonoxacin had good mycoplasmacidal activity against both MSMP and MRMP strains. The pharmacodynamic analysis demonstrated that the mycoplasmacidal effect of nemonoxacin was greater than those of moxifloxacin and levofloxacin based on the *K*_max_, displaying a dualism of concentration dependency in low minimum inhibitory concentrations (MICs) and time dependency in high MICs. Therefore, nemonoxacin demonstrated efficacy against *M. pneumoniae in vitro* and is a potential candidate for clinical studies for assessing its therapeutic effects against *M. pneumoniae* infections. The killing pattern or PK/PD characteristics of nemonoxacin against *M. pneumoniae* from *in vitro* data, animal studies, or clinical studies merit further evaluation for treating infections caused by the organism.

**IMPORTANCE:**

This study first reported the *in vitro* effector kinetics of the new non-fluorinated quinolone, nemonoxacin, against macrolide-resistant *M. pneumoniae* (MRMP) and macrolide susceptible *M. pneumoniae* (MSMP) strains along with other antimicrobial agents. The time-kill assays and pharmacodynamic analysis showed that nemonoxacin has significant mycoplasmacidal activity against MRMP and MSMP. This study paves the road to establish appropriate dosing protocols of a new antimicrobial drug for children infected with *M. pneumoniae*.

## INTRODUCTION

*Mycoplasma pneumoniae* belongs to the class *Mollicutes*, order *Mycoplasmatales*, and family *Mycoplasmataceae*, which is characterized by the lack of a cell wall (1). Because *M. pneumoniae* possesses a limited number of genes and low guanine and cytosine content and has simple metabolic pathways, it cannot synthesize some essential compounds (2). It is the causative agent of respiratory infections via the adhesion to the mucosal surface of the respiratory tract in children and adults (3). The pathogen can be transmitted directly through air and close contact; accordingly, the related infections are very common worldwide. Furthermore, the organism could result in extrapulmonary manifestations, such as neurological disorders, dermatological disorders, and nonspecific myalgias (1).

Developing vaccines for protecting against *M. pneumoniae* infection seemed promising in view of its rather homogeneous antigenicity (1). However, some studies showed that the protective efficacies were generally disappointing for a variety of reasons (1, 4, 5). As a result, no effective vaccine is available for preventing its infections at present (5). Therefore, antimicrobial chemotherapy still plays a significant role in treating *M. pneumoniae* infections. Because of the lack of a cell wall, the pathogen is naturally resistant to some agents targeting the cell wall, such as β-lactams, fosfomycin, and glycopeptides (3). Historically, the major antimicrobial classes against *M. pneumoniae* include macrolides and tetracyclines that act on the bacterial ribosome to inhibit protein synthesis and fluoroquinolones that inhibit DNA replication (3).

Owing to potential toxicities of tetracyclines and fluoroquinolones, macrolides are used as empirical agents for *M. pneumoniae* infections in children (3). However, an increasing number of studied reported on encounter with macrolide-resistant *M. pneumoniae* (MRMP) worldwide from the early 2000s (3). Particularly in some regions of China, the resistance rate of *M. pneumoniae* to macrolides was over 90% (6 – 8). Some studies reported that resistant strains can cause more severe or prolonged disease (3, 9, 10). Given the difficult situation of resistant *M. pneumoniae* infection, there is an urgent need for alternative treatments. Some studies have reported that quinolones (ciprofloxacin or levofloxacin) and tetracyclines (doxycycline or minocycline) may improve clinical efficacy after macrolide treatment failure in children (3, 11, 12). Tosufloxacin and tetracyclines were approved for pediatric use in patients with pneumonia who do not respond to macrolides in the Japanese guidelines for *M. pneumoniae* infections in 2014 (13). In consideration of potentially greater toxicity of tetracyclines in children, pediatricians in Japan frequently prescribe tosufloxacin for children with *Mycoplasma* infections (13). MRMP was effectively inhibited, and macrolide resistance rate was consecutively lowered after the application of tosufloxacin (13, 14). Furthermore, no tosufloxacin-resistant strains have currently been identified (13). Therefore, it is a great recommendation for advising clinicians to prescribe tosufloxacin to combat MRMP infections. However, in China, although quinolones and tetracyclines were also recommended as the second choice of treatment for MRMP infections in the Chinese guidelines for *M. pneumoniae* infections (2015), the macrolide resistance rate to date remains to be very high (6, 7, 15).

Nemonoxacin is a new C-8-methoxy non-fluorinated quinolone (NFQ) having broad-spectrum activity against gram-positive, gram-negative, and atypical pathogens *in vitro*, while showing general safety and good tolerance *in vivo* (16, 17). One recent study found that the efficacy of nemonoxacin (500 mg once daily for 7–10 days) is similar to that of levofloxacin for treating adult community-acquired pneumonia (CAP) in terms of clinical cure rates, microbiological success rates, and safety profile (18). In addition, Wang et al. reported that nemonoxacin possessed superior activity and bactericidal effects against *M. pneumoniae in vitro* in comparison with levofloxacin (19). Nemonoxacin was approved for CAP in adults in China in 2016. However, currently, there are no data showing the clinical efficacy of nemonoxacin against MRMP in children.

The culture process of *M. pneumoniae* is very slow, time-consuming, laborious, and expensive. Because of these reasons, static time-kill curves and pharmacodynamic analysis are rarely used to research agents against this organism. There is only one study in the recent past focusing on the bactericidal killing kinetics of clarithromycin, azithromycin, minocycline, doxycycline, and tosufloxacin against *M. pneumoniae* based on minimum inhibitory concentrations (MICs) or the peak serum concentrations observed in humans (20). However, this study did not include sufficient killing curves by using more drug concentrations and lacked further pharmacodynamic analysis. The relationship between drug concentrations and its pharmacological effect is often described by empirical mathematical models (21 – 24). The relationship is usually analyzed by the sigmoid *E*_max_ model. Therefore, the current study aimed to assess the killing kinetics of nemonoxacin and other antimicrobial agents against macrolide susceptible *M. pneumoniae* (MSMP) and MRMP by static time-kill curves analysis. In addition, the pharmacodynamic analysis of these drugs was performed using the sigmoid *E*_max_ model.

## MATERIALS AND METHODS

### Materials and *M. pneumoniae* culture

The *M. pneumoniae* reference strain ATCC 29342 and two clinical strains [strain 060411 (MSMP) and strain 071112 (MRMP)] were obtained from the strain bank at the Institute of Antibiotics, Huashan Hospital, Shanghai, China. The multiple-locus variable-number of tandem-repeats analysis (MLVA) of ATCC 29342 was M4-4-5-7-2. The two clinical strains also had the same MLVA type. As previously reported, a mycoplasma broth was prepared using mycoplasma broth base CM403 (Oxoid, Hampshire, United Kingdom), mycoplasma selective supplement G SR59 (Oxoid), 0.002% phenol red, and 0.5% glucose (25). Mycoplasma agar plates contained mycoplasma agar base CM401 (Oxoid) and mycoplasma selective supplement G SR59 (25). The broth inoculated with *M. pneumoniae* was incubated at 37°C aerobically for at least 3 days, whereas the agar plate was incubated at 37℃ with 5% CO_2_ for 6 weeks. The broth color shift from red to yellow without turbidity indicated *M. pneumoniae* growth. A color change with turbidity indicated bacterial and/or yeast contamination. The colonies of *M. pneumoniae* in agar plates were checked visually with the aid of a stereomicroscope.

### Antimicrobial susceptibility test (AST)

In this study, six antimicrobials were used. Nemonoxacin was provided by Zhejiang Medicine Co, Ltd, Zhejiang, China. Azithromycin, midecamycin, doxycycline, levofloxacin, and moxifloxacin were purchased from Dalian Meilun Biotech Co., China. MICs of the antimicrobials were determined by broth microdilution method according to the CLSI standards (26). In brief, the *M. pneumoniae* culture was diluted with mycoplasma broth to the desired inoculum concentration of 10^5^ CFU/mL. A series of concentrations of antimicrobial agents were prepared by twofold dilution with mycoplasma broth in a 96-well culture plate. Each well contained different concentrations of each antimicrobial and *M. pneumoniae* inoculum. A growth control (*M. pneumoniae* inoculum in the absence of antimicrobial agents) and a sterility control (sterility medium) were also included. After sealing the plates with a gas-permeable film, the plates were cultured at 37°C in an atmosphere with 5% CO_2_ (26).

### Static time-kill curves and analysis

Twofold dilution series of these six agents at final concentrations corresponding to multiples of the MICs were prepared in the mycoplasma broth. Similar to the MIC determination studies, *M. pneumoniae* was added to the mycoplasma broth with antimicrobials to obtain an initial inoculum of 10^5^ CFU/mL in 5 mL volumes. Growth controls (*M. pneumoniae* cultures in the absence of antimicrobials) were also included. Cultures were incubated for 5–6 days at 37°C in a humidified incubator with 5% CO_2_, and samples were taken at 0, 1, 2, 3, 4, 5, or 6 days. The cultures were then serially diluted 10-fold to 10^5^, and 100 µL aliquots of each dilution was transferred onto the solid agar plates. The plates were incubated for at least 14 days at 37°C in the humidified incubator with 5% CO_2_, and colonies were counted for the dilutions with 30–300 colonies per plate. Statistical analysis of the killing curves (i.e., the difference between log_10_ CFU per milliliter at *t* = 0 h and *t* = 120 h) was performed by paired *t*-tests. A *P* value of ≤ 0.05 was considered significant.

### Pharmacodynamic analysis

The kill rate can reflect the effect of antibacterial drugs against a pathogen. The greater the kill rate, the stronger the antibacterial effect of the drug. The colony counts were converted to CFU/mL and antimycoplasmal effect, which is defined as the difference in log_10_ (CFU/mL) units between 0 and 5 or 6 days, was calculated for each concentration of antimicrobial agents. The limit of detection was 100 CFU/mL. The time-kill curves of these agents against *M. pneumoniae* were presented by plotting log_10_CFU/mL against time (h) at different concentrations. Mycoplasmacidal activity was defined as 99.9% or 3 log_10_ (CFU/mL) unit reduction of mycoplasma. The growth data of the strains were fitted to the growth curves model described in equation (1) using Matlab2020b software depending on the bacteria.


(1) $$\frac{dM}{dt}=K_{net}\left(1-\frac{M}{M_{max}}\right)\left(1-e^{-\delta t}\right)M$$

where *t* is the time, *M* is the log_10_ (CFU/mL) units of *M. pneumoniae*, *K*_net_ is bacterial net growth rate constant, *M*_max_ is the maximum value of *M. pneumoniae* density, and *δ* is lag-phase effect factor; (1*−e^−δt^*) was used to adjust the lag-phase effect of *M. pneumoniae*. Using Matlab2020b software, the data were fitted to the sigmoid *E*_max_ model, described in equation (2) as follows:


(2) $$E_{max}=\frac{K_{max}C^{\gamma }}{EC_{50}^{\gamma }+C^{\gamma }}$$

where *E*_max_ is the maximum value of kill rate in a certain time interval, *K*_max_ is the maximum constant of kill rate, *EC_50_* is the concentration at which 50% of the maximal kill rate is achieved, *C* is the concentration of antimicrobial agent, and *γ* is the Hill coefficient, which reflects the steepness of the kill rate-concentration curve. Therefore, the density of *M. pneumoniae* varies over time as described in equation (3).


(3) $$\frac{dM}{dt}=\left[K_{net}\left(1-\frac{M}{M_{max}}\right)\left(1-e^{-\delta t}\right)-\frac{K_{max}C^{\gamma }}{EC_{50}^{\gamma }+C^{\gamma }}\right]M$$

Parametric data from different groups were compared via one-way ANOVA followed by the least significant difference (LSD) test. A *P* value of ≤ 0.05 was considered significant.

## RESULTS

### Determination of MICs and concentrations of time-kill assays

MICs and serial concentrations of the six antimicrobial against strains MP29342, 060411, and 071112 in time-kill assays are provided in Table 1. All three *M. pneumoniae* isolates were susceptible to the doxycycline and fluoroquinolones (levofloxacin and moxifloxacin) tested.

**TABLE 1:** Minimum inhibitory concentrations and serial concentrations of six agents against the three *Mycoplasma pneumoniae* stains^a^

	NEM		MFX		LVFX		DOX		AZM		MED	
Strains	MIC	Concentrations	MIC	Concentrations	MIC	Concentrations	MIC	Concentrations	MIC	Concentrations	MIC	Concentrations
MP29342	0.125	0.125–2 (1–16× MIC)	0.125	0.125–2 (1–16× MIC)	0.5	0.5–8 (1–16× MIC)	0.25	0.25–4 (1–16× MIC)	0.00025	0.00025–0.004 (1–16× MIC)	0.03	0.03–0.5 (1–16× MIC)
060411	0.125	0.125–2 (1–16× MIC)	0.125	0.125–2 (1–16× MIC)	0.5	0.5–8 (1–16× MIC)	0.25	0.25–4 (1–16× MIC)	0.0005	0.0005–0.008 (1–16× MIC)	0.06	0.06–32 (1–512× MIC)
071112	0.25	0.25–4 (1–16× MIC)	0.25	0.25–4 (1–16× MIC)	0.5	0.5–8 (1–16× MIC)	0.5	0.5–8 (1–16× MIC)	16	16–1,024 (1–64× MIC)	4	4–512 (1–128× MIC)

^a^ Unit: mg/L; NEM, nemonoxacin; MFX, moxifloxacin; LVFX, levofloxacin; DOX, doxycycline; AZM, azithromycin; MED, midecamycin.

The MICs of nemonoxacin for strains MP29342, 061114, and 071112 were 0.125 mg/L, 0.125 mg/L, and 0.25 mg/L, respectively. Nemonoxacin had a lower MIC than levofloxacin (0.5 mg/L for all strains) but had the same MIC as moxifloxacin. For the resistant strain 071112, the MIC of the 16-member macrolide midecamycin was lower than that of the 15-member macrolide azithromycin (4 mg/L vs 16 mg/L).

### Static time-kill curves

**Fig 1:**
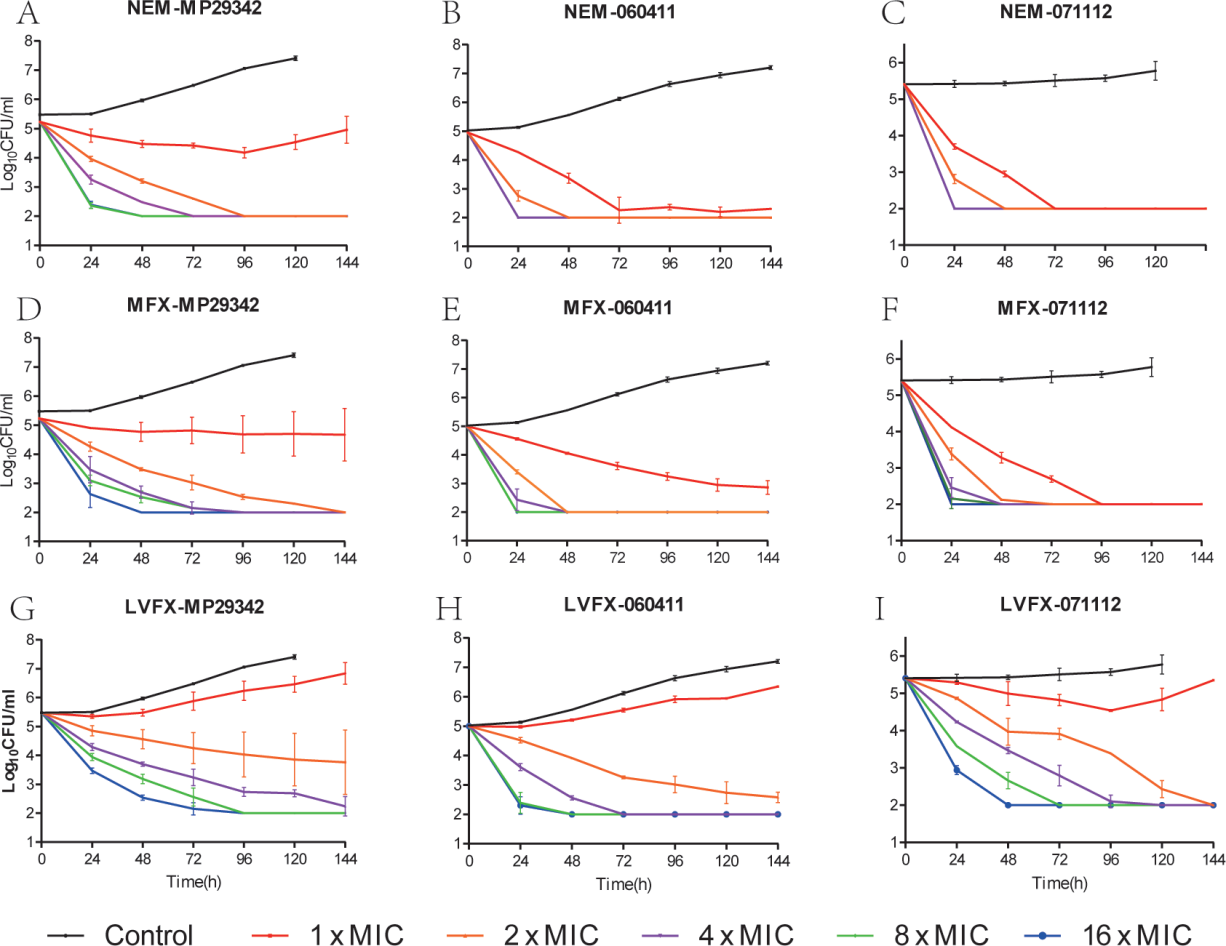
Time-kill curves of nemonoxacin (NEM), moxifloxacin (MFX), and levofloxacin (LVFX) against macrolide-susceptible *M. pneumoniae* (MSMP) strains MP29342 (A, D and G) and 060411 (B, E and H), as well as macrolide-resistant *M. pneumoniae* (MRMP) strain 071112 (C, F and I).

**Fig 2:**
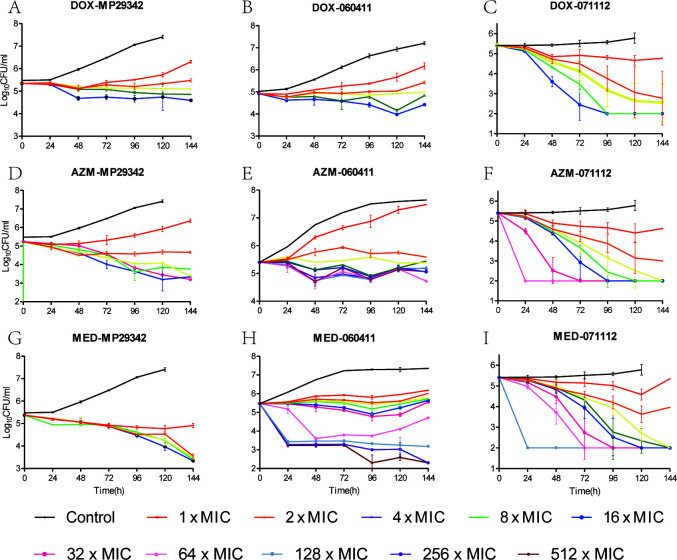
Time-kill curves of doxycycline (DOX), azithromycin (AZM), and midecamycin (MED) against macrolide-susceptible *M. pneumoniae* (MSMP) strains MP29342 (A, D and G) and 060411 (B, E and H), as well as macrolide-resistant *M. pneumoniae* (MRMP) strain 071112 (C, F and I).

The static time-kill curves of nemonoxacin, moxifloxacin, and levofloxacin are shown in Fig. 1. The static time-kill curves of doxycycline, azithromycin, and midecamycin are shown in Fig. 2. Within 6 days, the organism concentrations of MP29342, 060411, and 071112 in the blank-growth control group increased by 1.93 log_10_ CFU/mL (*P* < 0.05), 2.18 log_10_ CFU/mL (*P* < 0.05), and 0.37 log_10_ CFU/mL (*P* > 0.05), respectively.

Different serial concentrations of nemonoxacin resulted in different level of reductions in the growth of the three strains within six strains. When the drug concentration was at 1 × MIC, mild proliferation inhibition of the MP29342 strain was observed compared with the growth control group. There was a slow increase relative to the initial inoculum after Day 4. When the drug concentration was ≥2 × MIC, nemonoxacin showed bactericidal activity against MP29342 such that the organism concentrations were lower than the detection limits within 2–4 days. For strain 060411, when the drug concentration was at 1 × MIC, the organism concentration was decreased by 2.65 log_10_ CFU/mL (*P* < 0.05) within 6 days. At ≥2 × MIC drug concentration, the organism concentration decreased by at least 2.95 log_10_ CFU/mL within 1–2 days, achieving a mycoplasmacidal effect. For the macrolide- resistant strain 071112, when the drug concentration was ≥1 × MIC, the organism concentration showed a reduction of at least 3.40 log_10_CFU/mL within 1–3 days.

The static time-kill curves of moxifloxacin were similar to those of nemonoxacin for the three strains. For strain MP29342, mild proliferation inhibition at 1 × MIC was observed, but no regrowth was detected at a later time. When the drug concentration was ≥2 × MIC, moxifloxacin showed bactericidal activity against all strains with reductions of at least 3.23 log_10_ CFU/mL, 2.95 log_10_ CFU/mL, and 3.40 log_10_ CFU/mL in the concentration of strains MP29342, 060411, and 071112, respectively, within 1–6 days.

When the drug concentration was at 1 × MIC, levofloxacin presented little or no bacterial killing effect at any time, with all strains showing comparable growth to the controls across 6 days. With levofloxacin at ≥2 × MIC, bacterial killing of 2.34–3.47 log_10_ CFU/mL was observed across the first 2–4 days for strain MP29342, which was almost similar to the reduction observed for strains 060411 and 071112.

For the macrolide-susceptible strains MP29342 and 060411, doxycycline, azithromycin, and midecamycin resulted in little or no bacterial killing effects at 1×, 2×, 4×, 8×, and 16× MIC at any time. When the drug concentration was at 1× MIC, mild inhibition and subsequent regrowth of the three strains were observed across the 6 days. When the drug concentration was ≥2 × MIC, doxycycline showed bacteriostatic activity against strains MP29342 and 060411 within 6 days but did not show any bactericidal effect at all doses. There was also no obvious decline in the concentration of strain 060411 following azithromycin treatment within 6 days. Midecamycin produced no marked change in the organism concentration of strain 060411 within 6 days, except for a decrease at ≥64 MIC. For the macrolide-resistant strain 071112, doxycycline, azithromycin, and midecamycin showed growth reductions of 1.44–3.40 log_10_CFU/mL at 2×, 4×, 8×, and 16× MIC within 6 days. These data show that a higher concentration of antibiotic elicited better antibacterial effect.

### Pharmacodynamic analysis

The relationship between concentration and change in *M. pneumoniae* density was fitted by the *E*_max_ model, and the relative parameters are presented in Tables 2 and 3. The *K*_net_ values of the three strains were ranged from 0.001 to 0.0063, with strain MP29342 showing the best growth and strain 071112 showing the worst (*P* > 0.05). Similar to the results demonstrated in the time-kill curves, the *K*_max_ value of nemonoxacin derived from the *E*_max_ model was the highest (0.142). This verified that nemonoxacin had a better sterilizing effect than moxifloxacin (*P* < 0.05) and levofloxacin (*P* < 0.05) based on the *K*_max_ value. However, the *EC*_50_ of moxifloxacin was lower than that of nemonoxacin (*P* < 0.05) and levofloxacin (*P* < 0.05). Therefore, we tried to analyze the *K*_max_/*EC*_50_, wherein we found nemonoxacin or moxifloxacin was better than levofloxacin (*P* < 0.05). The *K*_max_/*EC*_50_ value of moxifloxacin (0.1) was higher than that of nemonoxacin (0.08), but it was not significant (*P* > 0.05).

**TABLE 2:** Parameter estimation derived from the *E*_max_ model^a^

Agents	Strains	*K*_net_	*M*_max_	*δ*	*K*_max_	*EC*_50_	*γ*
		(1 /h)	(Log_10_CFU/mL)	(1 /h)	(1 /h)	(mg/L)	\
NEM	MP29342	0.014	7.88	1.3003	0.136	2.34	0.91
	060411	0.011	7.92	1.1591	0.175	1.30	1.04
	071112	0.001	10.98	0.0811	0.115	1.53	1.09
MFX	MP29342	0.011	11.48	0.0186	0.024	0.26	1.77
	060411	0.007	11.46	0.0361	0.041	0.26	1.95
	071112	0.005	8.94	0.0055	0.044	0.55	1.43
LVFX	MP29342	0.063	12.79	0.0016	0.016	1.31	1.89
	060411	0.026	16.03	0.0030	0.030	1.61	2.35
	071112	0.020	14.33	0.0010	0.023	2.04	1.55

^a^ NEM, nemonoxacin; MFX, moxifloxacin; LVFX, levofloxacin; *K*_net_, the bacterial net growth rate constant; *M*_max_, the maximum value of *M. pneumoniae* density; *δ*, the lag-phase effect factor; *K*_max_, the maximum constant of kill rate; *EC*_50_, the concentration of the drug when the kill rate reaches 1/2 of *K*_max_ *; γ*, the Hill coefficient reflecting the steepness of the kill rate-concentration curve.

**TABLE 3:** Parameter estimation of different agents derived from the *E*_max_ model^*e*^

Agents	*K*_net_	*M*_max_	*δ*	*K*_max_	*EC*_50_	*K*_max_/*EC*_50_	*γ*
	(1 /h)	(Log_10_CFU/mL)	(1 /h)	(1 /h)	(mg/L)	\	\
NEM	0.009	8.93	0.847	0.142^*b*^	1.72	0.08	1.01
MFX	0.009	11.38	0.017	0.036	0.36^*c*^	0.1	1.71
LVFX	0.036^*a*^	14.38	0.002	0.023	1.65	0.01^*d*^	1.93

^*a*^ *P* < 0.05, compared to MFX.

^*b*^ *P* < 0.05, compared to MFX or LVFX.

^*c*^ *P* < 0.05, compared to NEM or LVFX.

^*d*^ *P* < 0.05, compared to NEM or MFX.

^*e*^ NEM, nemonoxacin; MFX, moxifloxacin; LVFX, levofloxacin; *K*_net_, the bacterial net growth rate constant; *M*_max_, the maximum value of *M. pneumoniae* density; *δ*, the lag-phase effect factor; *K*_max_, the maximum constant of kill rate; *EC*_50_, the concentration of the drug when the kill rate reaches 1/2 of *K*_max_ *; γ*, the Hill coefficient reflecting the steepness of the kill rate-concentration curve.

**Fig 3:**
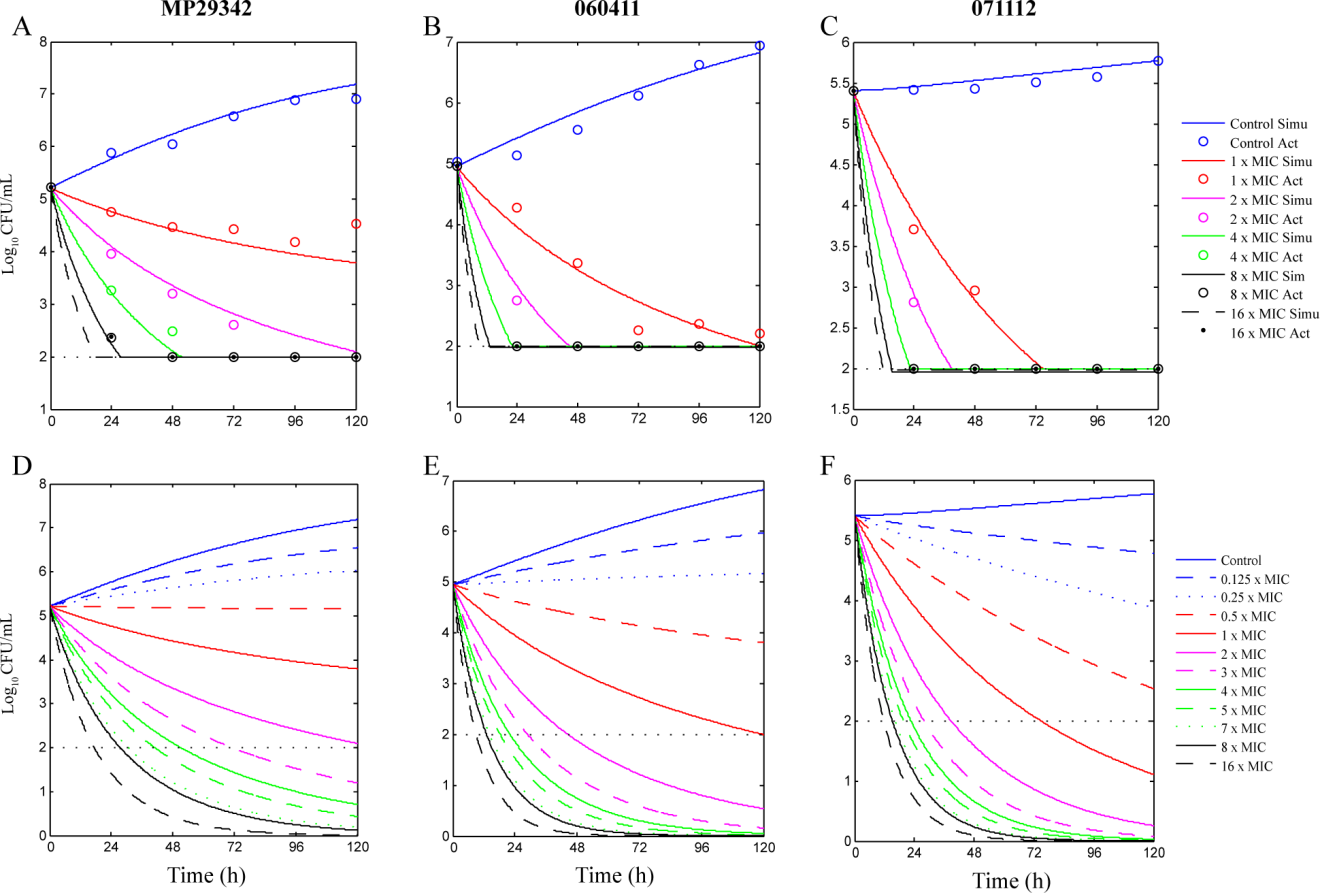
Best-fit curves (**A–C**) obtained from the *E*_max_ model and predicted curves (**D–F**) of *M. pneumoniae* strains exposed to nemonoxacin between 0 and 6 days. A and D, strain MP29342; B and E, strain 060411; C and F, strain 071112.

**Fig 4:**
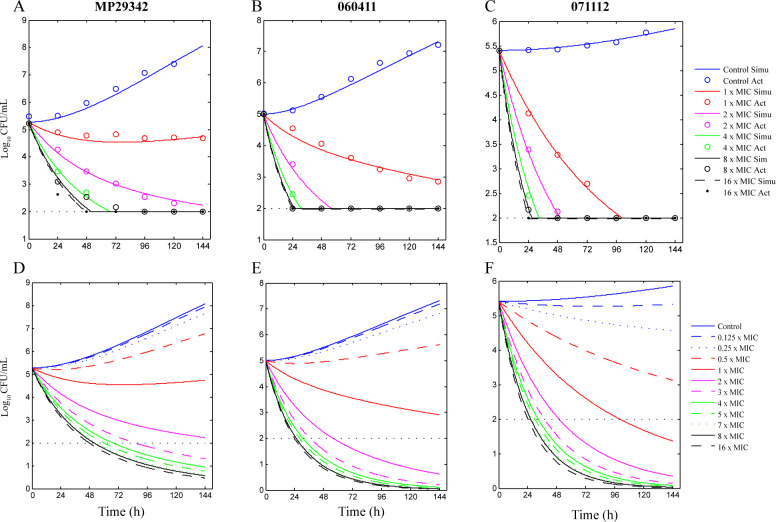
Best-fit curves (**A–C**) obtained from the *E*_max_ model and predicted curves (**D–F**) of *M. pneumoniae* strains exposed to moxifloxacin between 0 and 6 days. A and D, strain MP29342; B and E, strain 060411; C and F, strain 071112.

**Fig 5:**
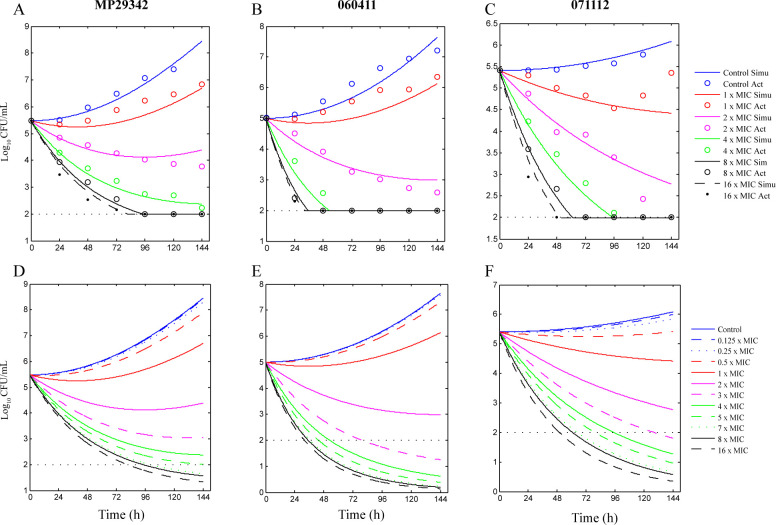
Best-fit curves (**A–C**) obtained from the *E*_max_ model and predicted curves (**D–F**) of *M. pneumoniae* strains exposed to levofloxacin between 0 and 6 days. A and D, MP29342; B and E, 060411; C and F, 071112.

The best-fit curves obtained from the *E*_max_ model and the predicted curves of *M. pneumoniae* strains exposed to nemonoxacin, moxifloxacin, and levofloxacin between Day 0 and 6 are described in Fig. 3 to 5. Model fitting values and measured values were uniformly distributed on both sides of the trend line. Strains MP29342, 060411, and 071112 all were slightly inhibited by nemonoxacin at 0.5× MIC, 0.125× MIC, and 0.125× MIC, respectively. For strain MP29342, the mycoplasmacidal effect of nemonoxacin was time-dependent at concentrations higher than 7× MIC. For strains 060411 and 071112, the time-dependent effect was at a concentration higher than 3× MIC. Below these concentrations, the mycoplasmacidal effect of nemonoxacin showed concentration dependence. Strains MP29342, 060411, and 071112 were slightly inhibited by moxifloxacin at MIC (0.125 mg/L), MIC (0.125 mg/L), 0.125× MIC (0.03125 mg/L), respectively. For strain MP29342, the mycoplasmacidal effect of moxifloxacin was time-dependent at concentrations higher than 5× MIC. For strains 060411 and 071112, a time-dependent effect was observed at concentrations higher than 3× MIC. Below these concentrations, the mycoplasmacidal effect of moxifloxacin showed concentration dependence. For levofloxacin, MP29342, 060411, and 071112 were slightly inhibited by at 2 × MIC, 2 × MIC, and 0.5 × MIC, respectively. For strain MP29342, the mycoplasmacidal effect of levofloxacin was time-dependent at concentrations higher than 7 × MIC. For strains 060411 and 071112, the time-dependent effect was at concentration higher than 4 × MIC and 5 × MIC, respectively. Below these concentrations, the mycoplasmacidal effect of levofloxacin showed concentration dependence.

## DISCUSSION

In this study, we reported the *in vitro* effector kinetics of the new NFQ nemonoxacin against MRMP and MSMP strains along with other antimicrobial agents. The time-kill assay and pharmacodynamic analysis showed that nemonoxacin had significant mycoplasmacidal activity against MRMP and MSMP.

*M. pneumoniae* is a pathogen that frequently causes pneumonia, which is often treated with macrolides (3). However, some strains have developed resistance to these drugs (3). Nemonoxacin is a new drug effective against *M. pneumoniae in vitro*, including strains resistant to macrolides (19). To date, few studies have evaluated the killing pattern or PK/PD characteristics of this drug against *M. pneumoniae* (3). Based on the methods for Antimicrobial Susceptibility Testing (AST) for *M. pneumoniae* described previously, the values of MIC observed in this study were within reasonable ranges (26). Consistent with the *in vitro* AST findings, the final inoculum of *M. pneumoniae* in each tube was approximately 10^5^ CFU/mL in the static time-kill experiment. This differs from approximately 10^6^ CFU/mL used in the study by Miyuki et al. (20). We previously detected the susceptibility of *M. pneumoniae*, *Ureaplasma* spp., and *M. hominis* to nemonoxacin and other drugs *in vitro* (19). We found that nemonoxacin was active against *M. pneumoniae* isolates with the lowest MIC_50_ (i.e., the minimal concentration that inhibits colony formation by 50%) and MIC_90_ (i.e., the minimal concentration that inhibits colony formation by 90%) values (0.125 and 0.25 mg/L, respectively) among all 11 drugs tested, and the dilutions were twofold lower than those of tetracyclines and levofloxacin (19).

To test the killing profiles, we further modeled the experimental data mathematically to provide an objective and quantitative evaluation. According to the static time-kill curves of quinolones and the corresponding predicted curves, we found that the quinolones resulted in a marked bactericidal effect against all strains. Higher concentrations of antibiotics showed a better antibacterial effect. The results of *E*_max_ model verified that the antibacterial effect of quinolones was concentration-dependent at concentrations lower than 16 × MIC, wherein the relationship between antimycoplasmal effect and PD parameters was fitted. With regard to the bactericidal characteristics of the antimicrobials, the antimicrobial agents can be classified into time- and concentration-dependent drugs. Although concentration-dependent drugs show faster killing with increasing concentrations, the kill rate of time-dependent drugs may be constant (27). Furthermore, the effect of antimicrobials is not static; instead, it is dependent on the interaction between the antibiotics and the bacteria (28). Identical agents can have different types of activities against various bacteria (29). Time-kill assays and subsequent pharmacodynamic analysis showed that nemonoxacin had significant mycoplasmacidal activity against MRMP and MSMP in this study. We also demonstrated that the mycoplasmacidal effect of nemonoxacin was greater than those of moxifloxacin and levofloxacin based on the *K*_max_ and that it showed dualism. The effect of nemonoxacin was mainly concentration dependent when the MIC was low. When the MIC was high, the mycoplasmacidal effect was time dependent. This characteristic was found in the classic fluoroquinolones such as levofloxacin and moxifloxacin, which has also been verified in other reports (30, 31). Our group previously investigated the PK/PD of nemonoxacin against *Streptococcus pneumoniae in vitro* using the *E*_max_ model to analyze the relationship between PK/PD parameters and drug effect (32). Similarly, we found dualism in the antibacterial effect of nemonoxacin against *Streptococcus pneumoniae*. Furthermore, we indicated that the regimens of 500 mg administered every 24 h and 750 mg every 24 h had favorable bacteriological efficacy and good clinical feasibility. The optimal dosing regimen mentioned above was supported and justified by the data on Chinese subjects receiving nemonoxacin for the treatment of community-acquired pneumonia caused by *Streptococcus pneumoniae* in phase I to III clinical trials (18, 33, 34). In conclusion, the killing pattern or PK/PD characteristics of nemonoxacin against *M. pneumoniae* from *in vitro* data, animal studies, or clinical studies merit further research for treating infections caused by the organism.

*M. pneumoniae* is one of the most important pathogen causing respiratory diseases in human (3). *In vitro* killing kinetics provide reference conditions for studying the pharmacodynamics of antimicrobial agents against *M. pneumoniae*. Although some antimicrobials are currently recommended for the treatment of respiratory diseases caused by *M. pneumoniae*, little work has been performed with regard to their killing kinetics (3, 20). Relevant studies have been hindered by several major difficulties. First, the culturing of this organism requires higher quality of nutrients and environment than that required for other bacteria. It must be cultured strictly in a rich and complex medium supplemented with a large amount of ingredients including great-quality serum (1, 35). Because of the characteristic of slow growth, experimental periodicity always needs longer time (26). Thus, it is very common for the culture to be contaminated by other microorganisms. Second, the culture medium is very expensive (28, 36, 37). Third, the present model of pharmacokinetics/ pharmacodynamics (PK/PD) for bacteria is not suitable for *M. pneumoniae* with conditions of small size (36). The *in vitro* model of PK/PD has only been applied to study drugs against fast-growing *Mycoplasma* species, such as *M. hyopneumoniae* and *M. gallisepticum* (28, 37, 38).

In this study, doxycycline, azithromycin, and midecamycin showed great antibacterial activity against MRMP as well as MSMP. Incremental doses of these three drugs resulted in marked killing of *M. pneumoniae* in the first 3 days. These results are consistent with previous reports in other organisms (38 – 41). Azithromycin has also been reported as a bactericidal agent against *Helicobacter pylori* (42). There was obvious regrowth of strain 060411 after being suppressed by these agents for 3 days. The three antibiotics showed better bactericidal activity against the macrolide-resistant strain 071112 compared with the macrolide-susceptible strains MP29342 and 060411, which may have a fitness cost because of the hampered protein synthesis. We have tested the growth curves of other MRMP strains (data not shown) and found that they all had a slower growth rate compared with the MSMP isolates. Our observation was different from the finding of Satoshi’s group, in which the growth of the MRMP strain was almost similar to that of the MSMP strain (20). On observing other microorganisms, slower growth of resistant pathogens was observed in many studies (43 – 49). Yuki et al. speculated that the slow growth of vancomycin-intermediate *Staphylococcus aureus* is owing to several mutations in genes involved in various metabolic pathways (50).

This study had two main limitations. First, the study was undertaken based on the *in vitro* static time-kill curves to evaluate PD. Although all experiments were performed laboriously and thoroughly, the PK and anti-inflammatory properties were not considered. This dynamic PK/PD model can provide a continuous flow of agent to simulate the PK in the human body. It also reflects the interaction between the drug and bacteria, quantifying the efficacy of the antimicrobial agents (51). Although there are challenges to apply the model to *M. pneumoniae*, future studies should attempte to conduct PK/PD experiments to resolve the issue of severe infections with MRMP. Second, only one clinical MRMP strain and one clinical MSMP strain were tested in this study, and all three strains share the same MLVA genotype. Considering the diversity of genotypes in *M. pneumoniae* clinical isolates, more strains should be tested to determine the general pharmacodynamic characteristics of *M. pneumoniae*.

The rate of MRMP infections is increasing worldwide and making treatment more difficult (3). Therefore, searching for alternative drugs against MRMP infections is an urgent requirement. It is superior to pursue pharmacokinetic parameters rather than MICs to better characterize new antibacterial agents. Our study provided useful basic information on the PK/PD experiments on *M. pneumoniae*, which will pave the way for establishing appropriate dosing protocols of a new antimicrobial drug for children infected with *M. pneumoniae*.
